# Minor GPI(-) granulocyte populations in aplastic anemia and healthy individuals derived from a few *PIGA*-mutated hematopoietic stem progenitor cells

**DOI:** 10.1038/s41408-023-00932-5

**Published:** 2023-11-08

**Authors:** Hiroki Mizumaki, Dung Cao Tran, Kohei Hosokawa, Kazuyoshi Hosomichi, Yoshitaka Zaimoku, Hiroyuki Takamatsu, Hirohito Yamazaki, Ken Ishiyama, Rena Yamazaki, Hiroshi Fujiwara, Atsushi Tajima, Shinji Nakao

**Affiliations:** 1 Department of Hematology, Faculty of Medicine, Institute of Medical Pharmaceutical and Health Sciences, Kanazawa University, Kanazawa, Japan; 2 Department of Bioinformatics and Genomics, Graduate School of Advanced Preventive Medical Sciences, Kanazawa University, Kanazawa, Japan; 3 Division of Transfusion Medicine, Kanazawa University Hospital, Kanazawa, Japan; 4 Department of Obstetrics and Gynecology, Graduate School of Medical Sciences, Kanazawa University, Kanazawa, Japan

**Keywords:** Haematopoietic stem cells, Genetics research, Haematological diseases

Dear Editor,

Glycosylphosphatidylinositol-anchored protein-deficient granulocytes (GPI[-] Gs) are often detected in the peripheral blood (PB) of patients with acquired aplastic anemia (AA) and are thought to represent immune pathophysiology of bone marrow failure [1–3]. Among these GPI(-) Gs, small paroxysmal nocturnal hemoglobinuria-type granulocyte (PNH-G) populations that account for 0.003%–1% of the total granulocytes, which we refer to as small-PNH-G populations in this manuscript, may differ from the larger (≥1.0%) PNH-G populations in terms of the clonal diversity and proliferative capacity of the X-linked phosphatidylinositol glycan class A gene (*PIGA*)-mutated cells that PNH-G populations originate from, because their percentages usually remain low for a long period of time [2]. In addition, a previous study demonstrated that GPI(-) Gs isolated from AA patients were a polyclonal population that had diverse mutations in the *PIGA* gene [4]. Conversely, some recent studies using deep next generation sequencing of the *PIGA* gene revealed that PNH-G populations ≥1.0% isolated from AA patients had a few different distinct *PIGA*-mutated sequences, suggesting that small-PNH-G populations may also be oligoclonal [5, 6]. However, the inability to precisely sequence *PIGA* in the small-PNH-G populations has hampered the evaluation of clonality.

Healthy individuals (HIs) are negative for small-PNH-G populations except in a few cases [7], but GPI(-) Gs <0.003%, which in this article are referred to as miniscule-PNH-G populations, may be detected in most HIs [8, 9]. Several studies demonstrated that GPI(-) Gs detected in HIs are short-lived polyclonal populations derived from *PIGA*-mutated committed progenitor cells rather than from hematopoietic stem and progenitor cells (HSPCs) with *PIGA* mutations [8, 9]. However, we previously identified two HIs with 0.01%–0.8% GPI(-) Gs that persisted several years at similar percentages, suggesting that miniscule-PNH-G populations in HIs might also be derived from long-lived HSPCs [7].

To address these issues, we first analyzed *PIGA* gene sequences in small-PNH-G populations isolated from five AA patients possessing 0.029%–0.810% GPI(-) Gs and three HIs who had been found to have 0.006%–0.059% GPI(-) Gs during a screening of more than 200 HIs for small-PNH-G populations [7], using *PIGA* deep amplicon sequencing (AmpliSeq) of GPI(-) Gs that were enriched with magnetic microbeads followed by flow cytometric cell sorting (Tables S1 and S2, Figs. S1 and S2). Genomic DNA from each sorted GPI(-) Gs and GPI(+) Gs was amplified using primers covering all *PIGA* exons and subjected to *PIGA*-AmpliSeq (Table S3). Details of materials and methods are provided in the Supplemental Data.

Our enrichment method that was developed by Araten et al. [8] enabled the detection of only 1–3 different *PIGA* mutations in all five AA patients (AA 1–5) (Table 1). Limited *PIGA* mutations were also detected in the three HIs (HI 1–3) (Fig. 1A, Table 1). A second *PIGA*-AmpliSeq performed one year after the first sequencing for HI 2 and HI 3 revealed that the same *PIGA* mutations persisted at similar allele frequencies (AFs) in each of the HIs (Fig. 1B, Table S4). For HI 1 and HI 2, the AFs of predominant *PIGA*-mutated sequences were longitudinally measured using whole-blood DNA samples with droplet digital PCR, which showed no apparent changes in the AF, 0.020%–0.027% for HI 1 and 0.012%–0.025% for HI 2 over three and six years, respectively (Fig. S3, Table S5).

**Table 1:** Somatic *PIGA* mutations identified in AA patients and healthy individuals.

Case	Gender	% of PNH-Gs at sampling	Type of Mutation	Region	Mutation (coding)	Mutation (protein)	Variant Allele Frequency (%)
AA 1	F	0.087	Frameshift deletion	Exon 6	c.1265delC	P422Qfs*1	49.4
AA 2	F	0.23	Missense	Exon 2	c.C44G	p.A15G	11.4
			Frameshift deletion	Exon 2	c.154delC	p.H52Tfs*8	5.1
			Frameshift deletion	Exon 6	c.1306_1307del	p.F436Pfs*15	14.9
AA 3	F	0.029	Missense	Exon 4	c.469G > C	p.A157P	42.3
AA 4	F	0.089	Frameshift insertion	Exon 2	c.196dupA	p.V67Gfs*62	39.5
			Splice site mutation	Intron 4	c.487-2A > G		4.1
AA 5	F	0.810	Missense	Exon 2	c.A154T	p.T172S	28.5
			Frameshift deletion	Exon 2	c.523_526del	p.L175Ffs*18	13.2
			Nonsense	Exon 6	c.G1331A	p.W444X	2.2
HI 1	M	0.051	Nonsense	Exon 3	c.718T > A	p.L271X	95.4
			Frameshift insertion	Exon 2	c.274dupT	p.L92Ffs*37	2.2
HI 2	F	0.059	Frameshift deletion	Exon 6	c.1280delT	p.I427Tfs*15	59.9
HI 3	M	0.006	Missense	Exon 2	c.C44G	p.A15G	85.7
			Non-frameshift deletion	Exon 5	c.1021_1032del	p.P341_L344del	4.4
			Frameshift deletion	Exon 2	c.154delC	p.H52Tfs*15	2.0
HI 4	M	0.000	Missense	Exon 2	c.353G > A	p.C183T	17.1
			Nonsense	Exon 4	c.979C > T	p.Q327X	99.6
HI 6	M	0.001	Missense	Exon 2	c.119A > T	p.D40V	14.7
			Missense	Exon 2	c.214C > T	p.H72Y	44.8
HI 11	F	0.001	Splice site mutation		c.487-1G > A		16.3
			Splice site mutation		c.849-1G > A		3.5
			Nonsense		c.1099A > T	p.K367X	3.2
HI 18	M	0.000	Missense	Exon 2	c.143G > A	p.G48D	13.0
			Nonsense	Exon 2	c.270T > G	p.Y90X	14.0
			Splice site mutation		c751 + 1G > A		38.7
CB 1	M	0.000	Missense	Exon 2	c.242G > A	p.C81Y	95.3

*PNH* paroxysmal nocturnal hemoglobinuria, *AA* aplastic anemia, *HI* healthy individual, *CB* cord blood, *F* Female, *M* Male.

**Fig. 1:**
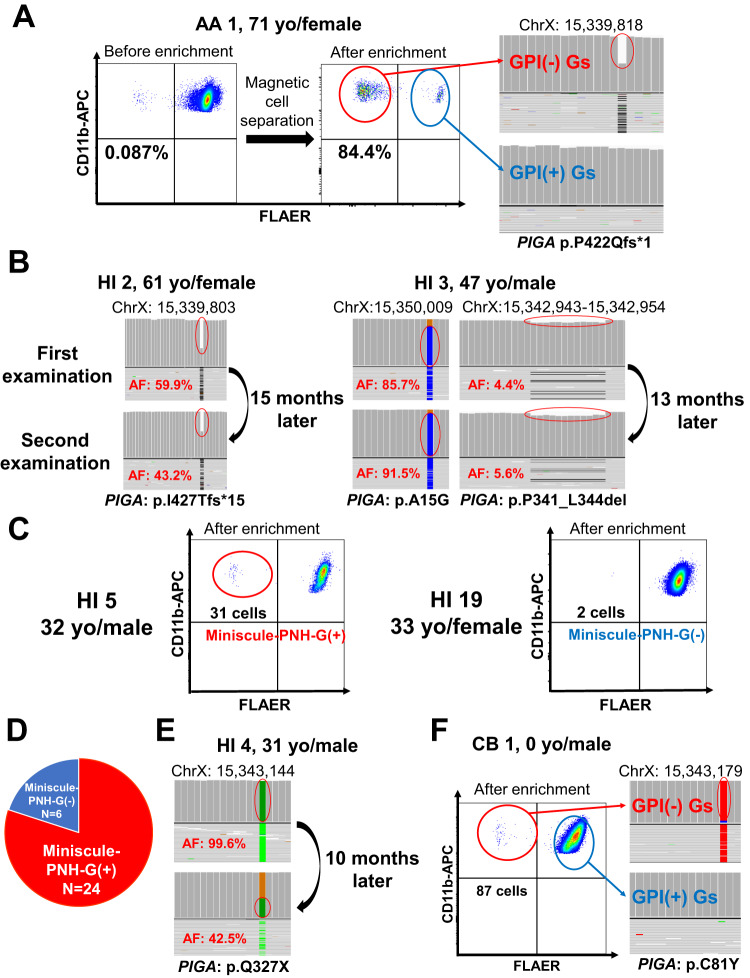
Detection of PNH-type granulocytes and *PIGA* mutations in AA patients and healthy individuals. **A** Representative flow cytometry plots of glycosylphosphatidylinositol-anchored protein-deficient granulocytes (GPI[-] Gs) in a patient with aplastic anemia (AA) after magnetic enrichment and a phosphatidylinositol glycan class A (*PIGA*) mutation in these GPI(-) Gs. 0.087% of GPI(-) Gs were enriched to 84.4% with the magnetic negative selection. A sufficient amount of DNA for *PIGA* amplicon sequencing (AmpliSeq) was obtained from sorted GPI(-) Gs. Integrative Genomics Viewer (IGV) showed a deletion mutation in GPI(-) Gs (circled in red). **B** Longitudinal analysis of *PIGA* mutations using *PIGA*-AmpliSeq of GPI(-) Gs in two healthy individuals (HIs) (HI 2 and HI 3). IGV showed three individual *PIGA* mutations in HI 2 and HI 3 at different time points (circled in red). **C** Representative flow cytometry plots of GPI(-) Gs after magnetic enrichment in HIs who had been judged to be negative for the presence of GPI(-) Gs by a high-sensitivity flow cytometry method. HI 5 was judged to be positive for miniscule paroxysmal nocturnal hemoglobinuria-type granulocyte (miniscule-PNH-G) populations and HI 19 was negative. **D** Proportions of HIs who possessed miniscule-PNH-G populations. Miniscule-PNH-G populations were detected in 24 (80%) of the 30 HIs. **E** Longitudinal analysis of *PIGA* mutations using *PIGA*-AmpliSeq of miniscule-PNH-G populations in HI 4. IGV showed the same *PIGA* nonsense mutation continuously detected at different time points (circled in red). **F** Flow cytometry plots of GPI(-) Gs in a cord blood (CB) sample (CB 1) and a *PIGA* mutation in these GPI(-) Gs. *PIGA*-AmpliSeq of GPI(-) Gs in CB 1 revealed a sole *PIGA* mutation (circled in red).

The presence of mono or oligoclonal GPI(-) Gs in the three HIs prompted us to study 30 HIs (male, *n* = 17, female, *n* = 13; median age, 37 [range, 27–65] years) and eight cord blood (CB) samples who had been judged to be negative (0%–0.002%) for small-PNH-G populations by a high-sensitivity flow cytometry [10]. The enrichment method identified a clear miniscule-PNH-G populations, which we defined as 10 or more CD11b^high^FLAER^-^ dots that formed a tight cluster, in 24 (80%) of the 30 HIs (Fig. 1C, D). The median number of GPI(-) Gs derived from 7 ml of PB from miniscule-PNH-G population(+) patients was 35 (range, 10–136) cells. Sufficient amounts of DNA for *PIGA*-AmpliSeq were obtained from sorted GPI(-) Gs of six out of 24 HIs. *PIGA*-AmpliSeq revealed 1–3 different *PIGA* mutations in four of the six subjects (Table 1). A second *PIGA-*AmpliSeq performed 10 and 7 months after the first sequencing for HI 4 and HI 6, respectively, detected the same nonsense mutation in miniscule-PNH-G populations of HI 4 that was detected by the first *PIGA*-AmpliSeq (Fig. 1E, Table S6). The examination of CB also revealed miniscule-PNH-G populations in four of eight different CB samples. *PIGA-*AmpliSeq of 87 GPI(-) Gs obtained from one male CB sample (CB 1) showed a sole *PIGA* mutation (Fig. 1F, Table 1).

Several studies demonstrated that unlike GPI(-) Gs from patients with florid PNH, small-PNH-G populations detected in AA patients consisted of cells with multiple *PIGA* mutations and postulated that some of the *PIGA*-mutated HSPCs were selected to grow due to secondary genetic changes, leading to hemolytic PNH [4, 5]. Although this hypothesis seems plausible, no evidence of such polyclonality in small-PNH-G populations has been demonstrated using the current sequencing technology. This study is the first to demonstrate that all PNH-G populations <1.0% in AA patients consist of one or a few *PIGA*-mutated clones. Our longitudinal *PIGA* analysis also clearly demonstrates that small-PNH-G populations in AA patients are derived from a limited number of *PIGA*-mutated HSPCs, a finding consistent with a previous report showing the larger (≥1.0%) PNH-G populations of AA patients arose from a few hematopoietic stem cells (HSCs) [11]. These findings suggest that the selection of *PIGA*-mutated HSCs by immune mechanisms, such as GPI-specific T cells [12], may occur at the time of early onset of AA, not in the transition from AA to PNH.

According to Dingli’s hypothesis, granulocytes derived from committed progenitor cells persist for up to 120 days [13]. A previous study identified miniscule-PNH-G populations with *PIGA* mutations at frequencies up to 0.005% in most HIs, which became undetectable two months later, except for one clone in a HI, which persisted for up to 164 days [8], suggesting that the vast majority of GPI(-) G populations <0.005% detected in HIs may be derived from *PIGA*-mutated committed myeloid progenitor cells rather than HSPCs. Hu et al. also concluded that PNH-G populations in HIs are all derived from committed progenitor cells by demonstrating that *PIGA*-mutated sequences in myeloid cells generated from proaerolysin-resistant colony-forming cells were highly diverse [9]. However, our study demonstrated that the same *PIGA*-mutated sequences detected in the minor PNH-G populations persisted for more than ten months. Therefore, the minor GPI(-) G populations possessed by HIs are thought to be clonal populations derived from a limited number of *PIGA*-mutated HSPCs. However, given that the persistence of the *PIGA*-mutated sequence was demonstrated only in one HI and the rest of the mutated sequences in the two miniscule-PNH-G samples obtained over 6 months apart from the same HI varied greatly, it is possible that *PIGA* mutation was sometimes in a bona fide stem cell and sometimes in a more downstream progenitor cells. As several X-linked genes, such as *GPA* and *XK*, share similarities with *PIGA* in that mutant phenotypes result in loss of specific proteins, which can be detected by flowcytometry [14, 15], analysis of the mutations in those genes using enriched mutant cells may help us to further understand mutation frequencies and the origin of mutant cells in HIs.

This study also identified miniscule-PNH-G populations in CB samples that were negative for small-PNH-G populations and single *PIGA* mutation in miniscule-PNH-G populations of one CB sample. Spencer et al. analyzed HSPCs in healthy fetuses using whole genome sequencing and revealed that HSPCs acquired around 40 somatic mutations by 18 weeks after conception [16]. Wong et al. analyzed somatic mutations associated with myeloid malignancies in 31 CB samples using error-corrected DNA sequencing and identified that 18% of CBs harbored somatic mutations with AFs of 0.2%–0.6% [17]. Our findings indicate that some HSPCs acquire somatic *PIGA* mutations during fetal development and that they minimally contribute to hematopoiesis.

This study has several limitations that need to be considered. First, as *PIGA* mutations were determined using a small amount of DNA extracted from isolated GPI(-) Gs, false-positive mutations might have occurred due to replication errors during amplification of the *PIGA* gene. Although a minimum cut-off value of 2% was used for variant allele calling in order to avoid false-positive mutation calling, we could not completely exclude false-positive results. Second, in contrast with false-positive mutations, some *PIGA* mutations with low AFs might be misclassified as negative and the number of *PIGA* mutations in miniscule-PNH-G populations could be underestimated. Nevertheless, our results clearly demonstrate that GPI(-) Gs of HIs have limited *PIGA* mutations with high AFs and some of GPI(-) Gs with the same *PIGA* mutations were continuously detected at different time points.

In conclusion, minor GPI(-) Gs detectable in AA patients and HIs are derived from a few *PIGA*-mutated HSPCs, not from committed myeloid progenitor cells, suggesting that the selection of *PIGA*-mutated HSPCs by immune mechanisms may occur at the time of early onset of AA, not in the transition from AA to PNH. Very small numbers of clonal GPI(-) Gs are present more frequently in HIs than previously thought and might also be derived from a few HSPCs with somatic *PIGA* mutations that occur during the fetal stage.

### Supplementary information

Supplemental data

## Data Availability

The data generated or analyzed during the current study are available from the corresponding author on reasonable request.
