# Supplementary material for: Minor GPI(-) granulocyte populations in aplastic anemia and healthy individuals derived from a few PIGA-mutated hematopoietic stem progenitor cells
Source: Blood Cancer J. 2023 Nov 8;13(1):165. doi: 10.1038/s41408-023-00932-5 (PMC10632376; doi:10.1038/s41408-023-00932-5)
Supplement: Supplementary file 1 — Supplemental data [file 41408_2023_932_MOESM1_ESM.docx]

**Supplemental information**

**Minor GPI(-) granulocyte populations in aplastic anemia and healthy individuals　derived from a few *PIGA*-mutated hematopoietic stem progenitor cells**

Hiroki Mizumaki^1^, Dung Cao Tran^1^, Kohei Hosokawa^1^, Kazuyoshi Hosomichi^2^, Yoshitaka Zaimoku^1^, Hiroyuki Takamatsu^1^, Hirohito Yamazaki^3^, Ken Ishiyama^1^, Rena Yamazaki^4^, Hiroshi Fujiwara^4^, Atsushi Tajima^2^ and Shinji Nakao^1^

^1^ Department of Hematology, Faculty of Medicine, Institute of Medical Pharmaceutical and Health Sciences, Kanazawa University, Kanazawa, Japan;

^2^ Department of Bioinformatics and Genomics, Graduate School of Advanced Preventive Medical Sciences, Kanazawa University, Kanazawa, Japan;

^3^ Division of Transfusion Medicine, Kanazawa University Hospital, Kanazawa, Japan;

^4^ Department of Obstetrics and Gynecology, Graduate School of Medical Science, Kanazawa University, Kanazawa, Japan;

Correspondence: Shinji Nakao, M.D, Ph.D.

Department of Hematology, Graduate School of Medical Sciences, Kanazawa University, 13-1 Takaramachi, Kanazawa, Ishikawa, 920-8640, Japan.

E-mail: snakao8205@staff.kanazawa-u.ac.jp

Phone: +81-76-265-2274

FAX: +81-76-234-4252

**SUPPLEMENTAL METHODS**

Definition of aplastic anemia severity

Enrichment of minor GPI(-) granulocytes with magnetic microbeads followed by flow cytometry cell sorting

Droplet digital PCR assay for detecting *PIGA mutations* in HI 1 and HI 2

Next generation sequencing of the *PIGA* gene

Ethics approval statement

References

**SUPPLEMENTAL TABLES**

Supplemental Table 1: Characteristics of AA patients and HIs with small PNH-type granulocyte populations

Supplemental Table 2. The monoclonal antibodies used for magnetic and flow cytometric sorting

Supplemental Table 3. Sequence of primers used for amplification of the *PIGA* gene

Supplemental Table 4. Somatic *PIGA* mutations identified in HI 2 and HI 3 by second *PIGA* amplicon sequencing

Supplemental Table 5. Sequence of primers and probes using for droplet digital PCR assay

Supplemental Table 6. Somatic *PIGA* mutations identified in HI 4 and HI 6 by second *PIGA* amplicon sequencing

**SUPPLEMENTAL FIGURES**

Supplemental Figure 1. A workflow diagram

Supplemental Figure 2. A gating strategy for flowcytometric sorting of GPI(-) granulocytes

Supplemental Figure 3. Longitudinal droplet digital PCR analysis of *PIGA* mutations in HI 1 and HI 2

**SUPPLEMENTAL METHODS**

**Definition of aplastic anemia severity**

Severe aplastic anemia (AA) was diagnosed when at least two of the following criteria were met; the neutrophil count was < 0.5 × 10^9^/L, the platelet count was < 20 × 10^9^/L and the reticulocyte count was < 20 × 10^9^/L. Very severe AA was defined as a neutrophil count < 0.2 × 10^9^/L in addition to the criteria for severe AA. Non-severe AA defined as AA that does not meet the diagnostic criteria of severe AA.^1^ The response criteria have been described previously.^2^

**Enrichment of minor GPI(-) granulocytes with magnetic microbeads followed by flow cytometry cell sorting**

Leukocytes from subjects were treated with PE-labeled anti-CD55 monoclonal antibodies (mAbs) and anti-CD59 mAbs, and CD55^+^CD59^+^ granulocytes were removed using magnetic microbeads labeled with anti-PE mAbs (Anti-PE MicroBeads, Miltenyi Biotec, Gaithersburg, MD, USA) (Figure S1). Paired fractions of CD11b^+^FLAER-negative granulocytes (GPI[-] Gs) and CD11b^+^FLAER-positive granulocytes (GPI[+] Gs) were sorted from granulocytes using a fluorescence-activated cell sorter (BD FACSAria™ Fusion, BD Biosciences, Franklin Lakes, NJ, USA) (Figure S2, Table S2).

**Droplet digital PCR assay for detecting *PIGA mutations in* HI 1 and HI 2**

We designed droplet digital PCR (ddPCR) assays to detecting individual phosphatidylinositol glycan class A gene (*PIGA*) mutations in peripheral blood of healthy individual (HI) 1 and HI 2 using a QX200 AutoDG Droplet Digital PCR System (Bio-Rad Laboratories, Hercules, CA, USA), according to the manufacturer`s instructions. Briefly, we designed two different sets of primer pairs complementary to the consensus sequences of *PIGA* gene, and locked nucleic acid (LNA)-based probes with a non-fluorescent quencher (Iowa Black^®^FQ[IBFQ]**)** complementary to wild-type (WT) and mutant-specific (MT) sequences, which were labeled with different fluorochromes (hexachlorofluorescein [HEX] for WT and carboxyfluoresein [FAM] for MT) using PrimerQuest and BioPhysics (Integrated DNA Technologies, Coralville, IA, USA) (Table S5). The reaction mixtures for ddPCR consisted of the 2 LNA probes, 1 set of the primer pairs and 100 ng of genomic DNA, 5 μl of 4×ddPCR Multiplex Supermix (Bio-Rad), and deionized water in a final volume of 20 μl. Cycling conditions were as follows; 95°C/10 min for enzyme activation, followed by 40 cycles of 94°C/30 sec and 59°C/1 min, and 98°C/10 min for enzyme deactivation. Droplets were measured with the QX200 droplet reader and were analyzed with Quantasoft software ver 1.7.4 (Bio-Rad), according to the manufacturer’s instructions. The fractional abundance of the mutant allele was obtained by dividing the number of copies of the mutant allele (FAM) by the total number of copies of the WT allele (HEX) plus the mutant allele (FAM). Results were considered evaluable when the number of accepted droplets per well was at least 10,000. The detection limit of each *PIGA*-mutated sequence was found to be 0.01%.

**Next generation sequencing of the *PIGA* gene**

Nucleotide sequences of *PIGA* in sorted granulocytes of the AA patients, HIs and cord bloods were determined by deep sequencing of long-range PCR amplicons of *PIGA* with a next generation sequencer (MiSeq; illumina, San Diego, CA, USA). Genomic DNA from glycosylphosphatidylinositol-anchored protein-deficient granulocytes (GPI[-] Gs) and wild-type granulocytes was extracted using NucleoSpin^®^ Tissue XS (Takara bio, Shiga, Japan). Each exons of *PIGA* gene was individually amplified in the PCR mixtures containing 0.5 to 10 ng of genomic DNA, 0.2 μM of primers in 20 μl of 1 × PrimeSTAR GXL DNA Polymerase (Takara Bio). The primer sequences are shown in supplemental Table 2. Cycling conditions were as follows; 35 cycles of 98°C for 10 sec, 60°C for 15 sec, and 68°C for 3 min. Amplicons of each *PIGA* exons was mixed and subjected to library construction for *PIGA* amplicon sequencing (AmpliSeq). Paired-end sequence reads (150 bp read 1 and 151 bp read 2 in length) were obtained by using the MiSeq sequencer. Somatic mutations were detected as difference from reference sequence with allele frequency (AF <2%), and covering with at least 1000 reads. The median read depths for *PIGA*-AmpliSeq in the subjects were 5,021 (801-13,233). Paired-end illumine reads were mapped to the reference genome (GRCh37) using Burrows-Wheeler Aligner (bwa) v.0.7.17.^3^ bwa-generated SAM files were converted to the BAM format, then sorted and indexed using SAM tools v.1.9.^4^ Duplicated reads were marked with Picard v.2.26　(https://github.com/broadinstitute/picard) . After alignment of reads, the heuristic somatic mutation caller, VarScan 2, was used to detect somatic mutation.^5^ The mutations were reviewed using Unified Genotyper in the Genome Analysis Toolkit (GATK) v4.18 and the alignment data from granulocytes were visually compared via Integrative Genomics Viewer (IGV).^6, 7^ The somatic mutations were given functional annotation and COSMIC (https://cancer.sanger.ac.uk/cosmic) mutation ID meaning by ANNOVAR.^8^

**Ethics approval statement**

This retrospective study was performed in accordance with the Declaration of Helsinki. This study protocol was approved by the ethics committee of the Kanazawa University, Institute of Medical, Pharmaceutical, and Health Sciences (approval no. 2020-005, 2021-023).

**References**

1. Bacigalupo A, Hows J, Gluckman E, Nissen C, Marsh J, Van Lint MT*, et al.* Bone marrow transplantation (BMT) versus immunosuppression for the treatment of severe aplastic anaemia (SAA): a report of the EBMT SAA working party. *Br J Haematol* 1988 Oct; **70**(2)**:** 177-182.

2. Camitta BM. What is the definition of cure for aplastic anemia? *Acta Haematol* 2000; **103**(1)**:** 16-18.

3. Li H, Durbin R. Fast and accurate short read alignment with Burrows-Wheeler transform. *Bioinformatics* 2009 Jul 15; **25**(14)**:** 1754-1760.

4. Li H, Handsaker B, Wysoker A, Fennell T, Ruan J, Homer N*, et al.* The Sequence Alignment/Map format and SAMtools. *Bioinformatics* 2009 Aug 15; **25**(16)**:** 2078-2079.

5. Koboldt DC, Zhang Q, Larson DE, Shen D, McLellan MD, Lin L*, et al.* VarScan 2: somatic mutation and copy number alteration discovery in cancer by exome sequencing. *Genome Res* 2012 Mar; **22**(3)**:** 568-576.

6. McKenna A, Hanna M, Banks E, Sivachenko A, Cibulskis K, Kernytsky A*, et al.* The Genome Analysis Toolkit: a MapReduce framework for analyzing next-generation DNA sequencing data. *Genome Res* 2010 Sep; **20**(9)**:** 1297-1303.

7. Robinson JT, Thorvaldsdottir H, Winckler W, Guttman M, Lander ES, Getz G*, et al.* Integrative genomics viewer. *Nat Biotechnol* 2011 Jan; **29**(1)**:** 24-26.

8. Wang K, Li M, Hakonarson H. ANNOVAR: functional annotation of genetic variants from high-throughput sequencing data. *Nucleic Acids Res* 2010 Sep; **38**(16)**:** e164.

**SUPPLEMENTAL TABLES**

**Supplemental Table 1: Characteristics of AA patients and HIs with small PNH-type granulocyte populations**

| **Case** | **Age** | **Gender** | **Severity** | **Treatment** | **Time after IST, year** | **Response to IST at sampling** |
| --- | --- | --- | --- | --- | --- | --- |
| AA 1 | 71 | F | NSAA | rATG+CsA | 13 | PR |
| AA 2 | 74 | F | NSAA | CSA | 10 | PR |
| AA 3 | 66 | F | SAA | rATG+CsA | 5 | PR |
| AA 4 | 64 | F | NSAA | CsA+Romi | 2 | CR |
| AA 5 | 74 | F | NSAA | rATG+CsA+EPAG | 7 | PR |
| HI 1 | 71 | M | N/A | N/A | N/A | N/A |
| HI 2 | 61 | F | N/A | N/A | N/A | N/A |
| HI 3 | 47 | M | N/A | N/A | N/A | N/A |

Abbreviations: AA, Aplastic anemia; HI, Healthy individual; F, Female; M, Male; NSAA, Non-Severe Aplastic Anemia; SAA, Severe Aplastic Anemia; N/A, not applicable; rATG, rabbit antithymocyte globulin; CsA, Cyclosporine A; Romi, Romiplostim; EPAG, Eltrombopag; IST, Immunosuppressive therapy; PR, Partial Response; CR, Complete response.

**Supplemental Table 2.** **The monoclonal antibodies used for magnetic and flow cytometric sorting**

| Antigen | Isotype | Conjugate | Source | Cat No |
| --- | --- | --- | --- | --- |
| CD55 | Mouse IgG2a | PE | BD Biosciences | 555694 |
| CD59 | Mouse IgG2a | PE | BD Biosciences | 555764 |
| CD45 | Mouse IgG1 | PE-Cy7 | BD Biosciences | 557748 |
| CD11b | Mouse IgG2b | APC | Biolegend | 101212 |
| 7-AAD | Mouse IgG2b | N/A | BD Biosciences | 559925 |
| FLAER | Mouse IgM | Alexa Flour® 488 | Pinewood | FL2S-R |
| UltraPure MicroBeads | Mouse IgG1 | PE | Miltenyi Biotec | 130-105-639 |

Abbreviations: 7-AAD, 7-Amino-Actinomycin D; FLAER, fluorescent-labeled inactive toxin aerolysin; PE, phycoerythrin; PE-Cy7, phycoerythrin-Cy7 tandem; APC, allophycocyanin; N/A, not applicable; Cat No, Catalog Number.

**Supplemental Table 3: Sequence of primers used for amplification of the *PIGA* gene**

| **Target region** | **Description** | **Sequence（5’→3’）** |
| --- | --- | --- |
| *PIGA* exon 1 | Forward Primer | GAGGAGGGAGAATGCAATCAGGCAA |
|  | Reverse Primer | ACTCTCGGAAAGACCCCAGAAGTGT |
| *PIGA* exon 2 | Forward Primer | GCTAAACCAGGTCCAAATGCAACGG |
|  | Reverse Primer | AAAGCCATCTGTGAGCTCTGCCC |
| *PIGA* exon 3-5 | Forward Primer | GCCTGCTCTGGAGAAAACGAACTCA |
|  | Reverse Primer | AAGAGGCATGAGGCAGAGAACATCA |
| *PIGA* exon 6 | Forward Primer | GTGACTCCCTAGGGGAGAGGAACTG |
|  | Reverse Primer | AGGAAACAAAGGCACAATTGAACTCGC |

Abbreviations: *PIGA*, phosphatidylinositol glycan class A.

**Supplemental Table 4. Somatic *PIGA* mutations identified in HI 2 and HI 3 by second *PIGA* amplicon sequencing**

| Case | Type of Mutation | Region | Mutation (coding) | Mutation (protein) | Allele Frequency (%) |
| --- | --- | --- | --- | --- | --- |
| HI 2  (15 months after first AmpliSeq) | Frameshift deletion | Exon 6 | c.1280delT | p.I427Tfs*15 | 43.2 |
| HI 3  (13 months after first AmpliSeq) | Missense | Exon 2 | c.C44G | p.A15G | 91.5 |
|  | Non-Frameshift deletion | Exon 5 | c.1021_1032del | p.P341_L344del | 5.6 |
|  | Frameshift  deletion | Exon 3 | c.215delT | p.L72Rfs*23 | 4.0 |
|  | Splice site mutation |  | c.486+1C>T |  | 9.8 |

Abbreviations: AmpliSeq, Amplicon sequencing; HI, healthy individual.

Red letters indicate *PIGA* mutations which were identified in each of HIs by first *PIGA*-AmpliSeq.

**Supplemental Table 5.** **Sequence of primers and probes using for droplet digital PCR assay**

| **Name** | **Description** | **Sequence（5’→3’）** |
| --- | --- | --- |
| Primer set for HI 1 | Forward Primer | TAATTGGAGGAGAGGGACC |
|  | Reverse Primer | TCTCCCTCAAGACAACATGAA |
| Probe set for HI 1 | LNA probe for WT (HEX) | CATT+T+T+GGA+A+GA+AGT |
|  | LNA probe for MT (FAM) | CATT+T+A+GGA+A+GA+AGT |
| Primer set for HI 2 | Forward Primer | CAGACTTATTTCTCACTGCGG |
|  | Reverse Primer | CAGTGGCATCTATTGCAACAT |
| Probe set for HI 2 | LNA probe for WT (HEX) | CA+AA+G+A+TGTA+GC+CT |
|  | LNA probe for MT (FAM) | CA+AA+G+T+GT+AG+CC |

Abbreviations: HI, healthy individual; LNA, locked nucleic acid; FAM, carboxyfluoresein; HEX, hexachlorofluorescein; WT, wild-type specific sequences; MT, mutant-specific sequences.

+C, +G, and +T indicates bases which connect to LNA.

**Supplemental Table 6. Somatic *PIGA* mutations identified in HI 4 and HI 6 by second *PIGA* amplicon sequencing**

| Case | Type of Mutation | Region | Mutation (coding) | Mutation (protein) | Allele Frequency (%) |
| --- | --- | --- | --- | --- | --- |
| HI 4  (10 months after first AmpliSeq) | Nonsense | Exon 4 | c.979C>T | p.Q327X | 42.5 |
|  | Frameshift deletion | Exon 5 | c.1151delT | p.F384Sfs*40 | 52.7 |
|  | Frameshift deletion | Exon 6 | c.1308_1311del | p.L437Sfs*5 | 30.4 |
| HI 6  (7 months after first AmpliSeq) | Nonsense | Exon 3 | c.754G>A | p.Q252X | 18.6 |
|  | Nonsense | Exon 3 | c.762T>G | p.Y254X | 11.4 |

Abbreviations: HI, healthy individual; AmpliSeq, Amplicon sequencing.

Red letters indicate a *PIGA* mutation which was identified in HI 4 by first *PIGA*-AmpliSeq.

**SUPPLEMENTAL Figures**

**Supplemental Figure 1. A workflow diagram**


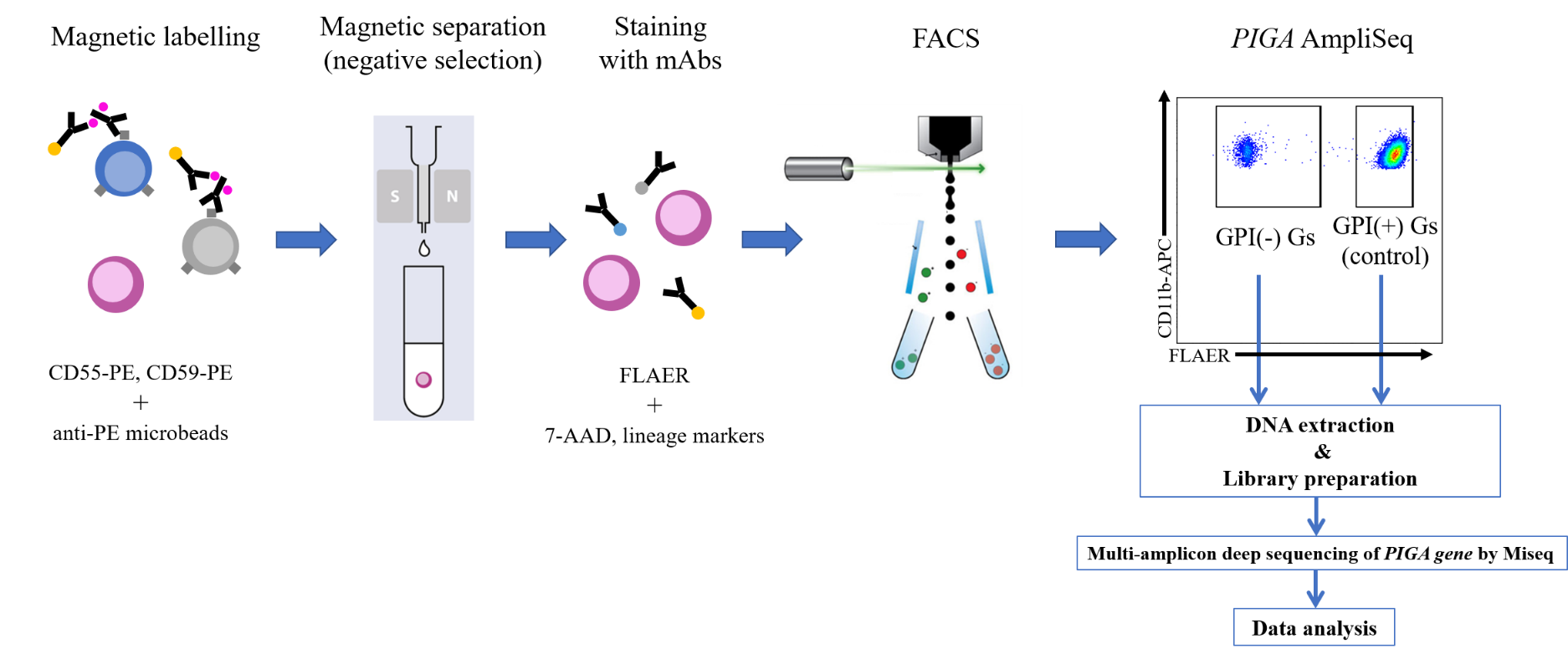
Peripheral blood leukocytes were enriched with glycosylphosphatidylinositol-anchored protein-deficient granulocytes (GPI[-] Gs) using negative immunomagnetic selection, and paired fractions of GPI(-) Gs and GPI(+) Gs were sorted using a fluorescence-activated cell sorter (FACSAria fusion). DNA from sorted granulocytes was amplified using primers covering all exons of *PIGA* and subjected to *PIGA* amplicon sequencing (AmpliSeq) with a next generation sequencer (Miseq).

**Supplemental Figure 2.** **A gating strategy for flowcytometric sorting of GPI(-) granulocytes**


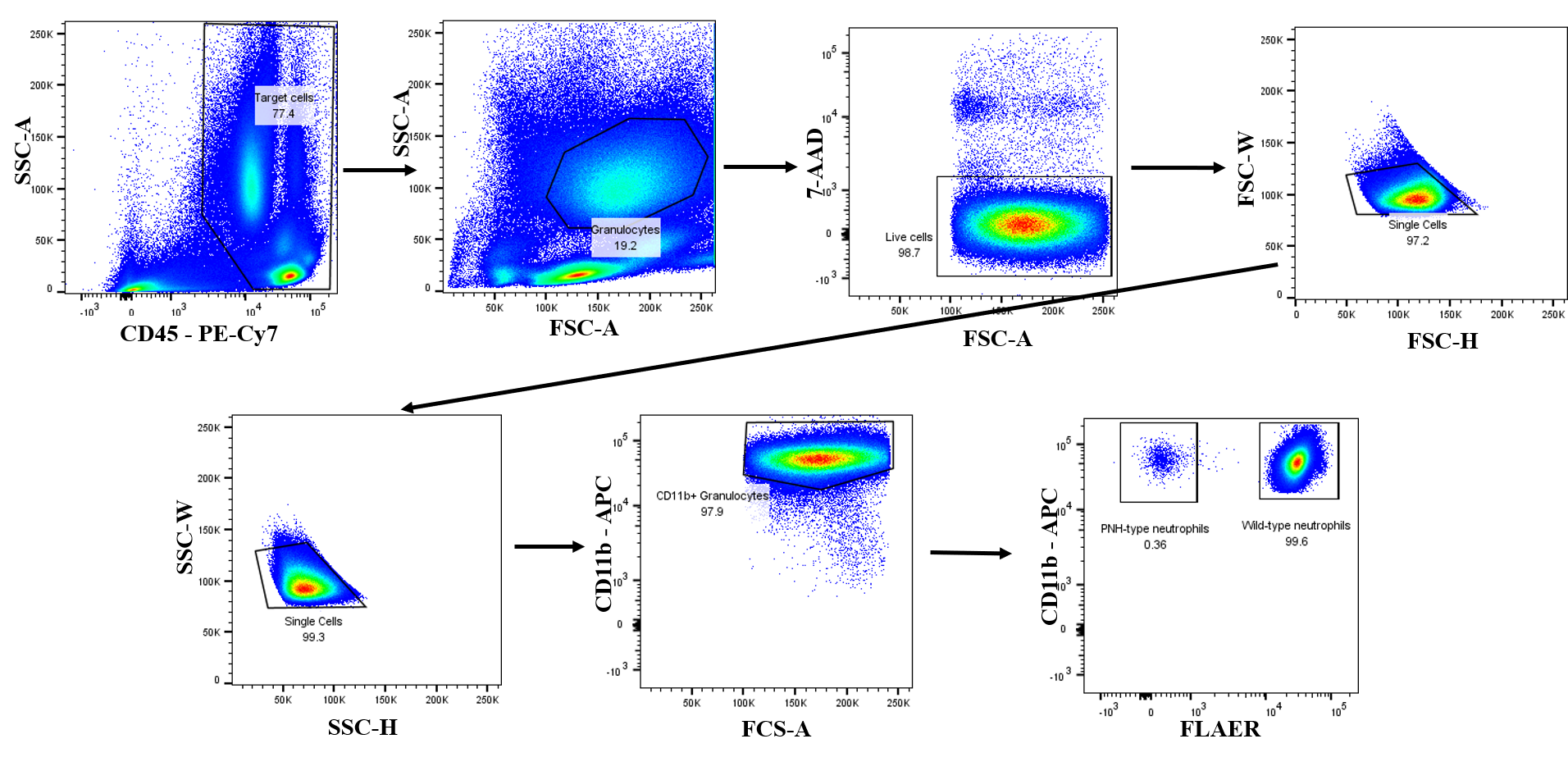


Peripheral blood samples were stained with antibodies (shown in the Supplementary Table 1). Target cell populations were gated in the following order; 1. CD45^+^ blood cells, 2. High FSC-A and high SSC-A cells that correspond to the granulocyte fraction, 3. 7-AAD^-^ viable granulocytes, 4. Single granulocyte fraction excluding doublet granulocytes, 5. CD11b^+^ granulocytes, 6. CD11b^+^FLAER^-^ (GPI[-] Gs) and CD11b^+^FLAER^+^ granulocytes (GPI[+] Gs).

**Supplemental Figure 3. Longitudinal droplet digital PCR analysis of *PIGA* mutations in HI 1 and HI 2**


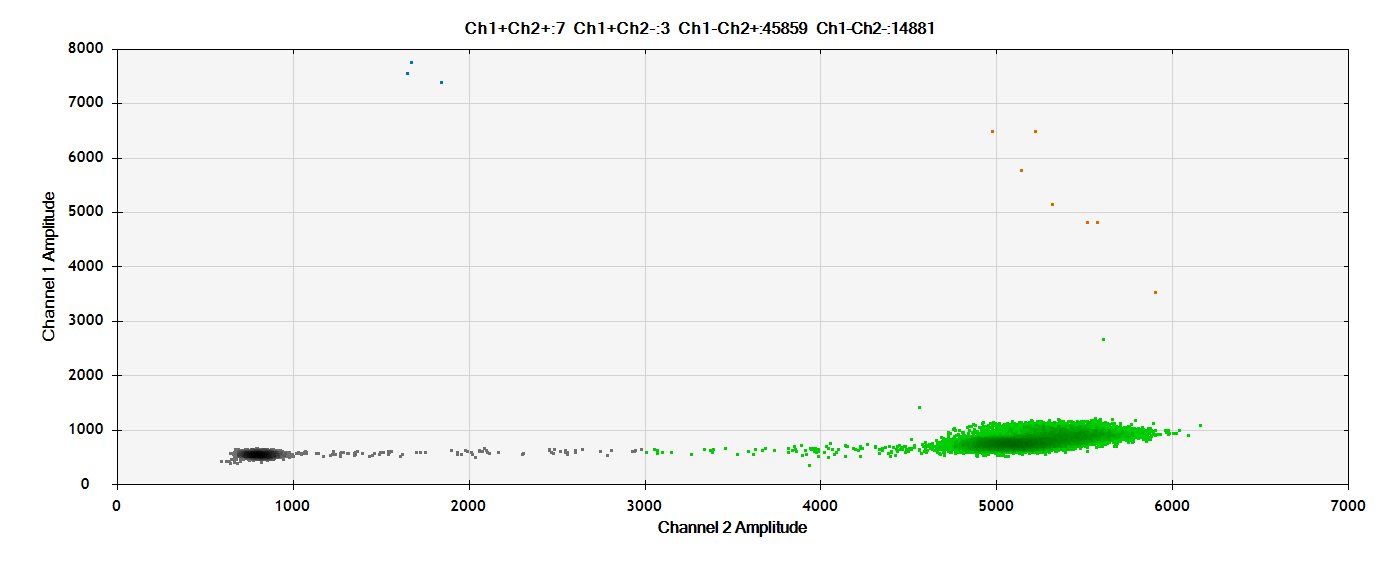

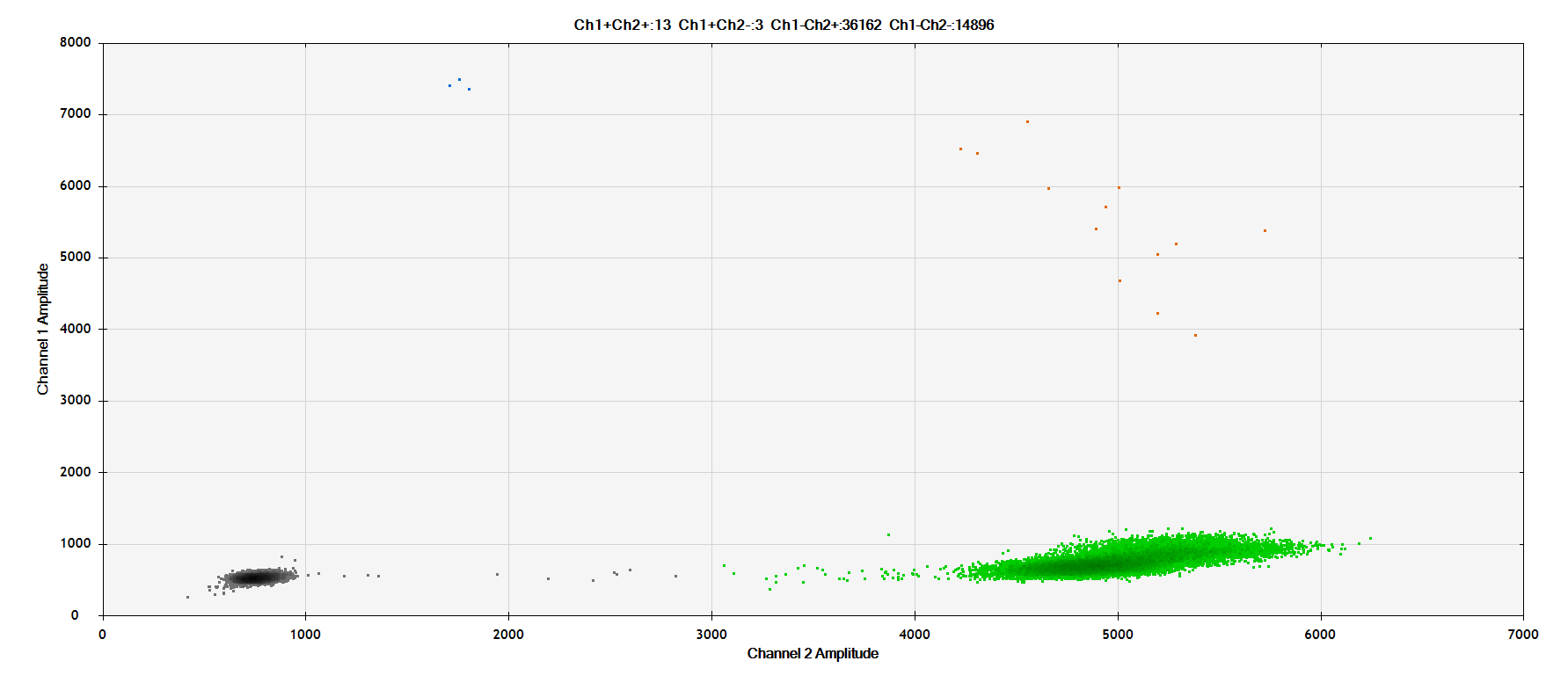


AF=0.012%

6 / X (month / year)

AF=0.025%

FAM (Mt)

HEX (Wt)

8 / X+6


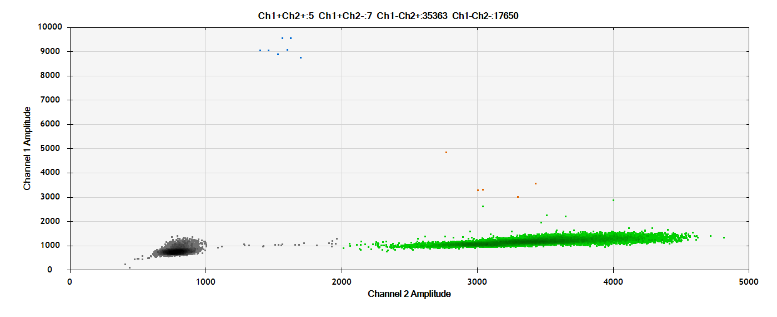


AF=0.020%

12 / X (month/ year)


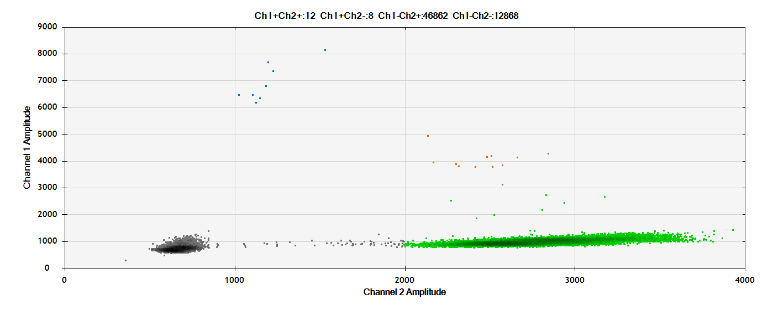


6 / X+1

AF=0.027%

FAM (Mt)


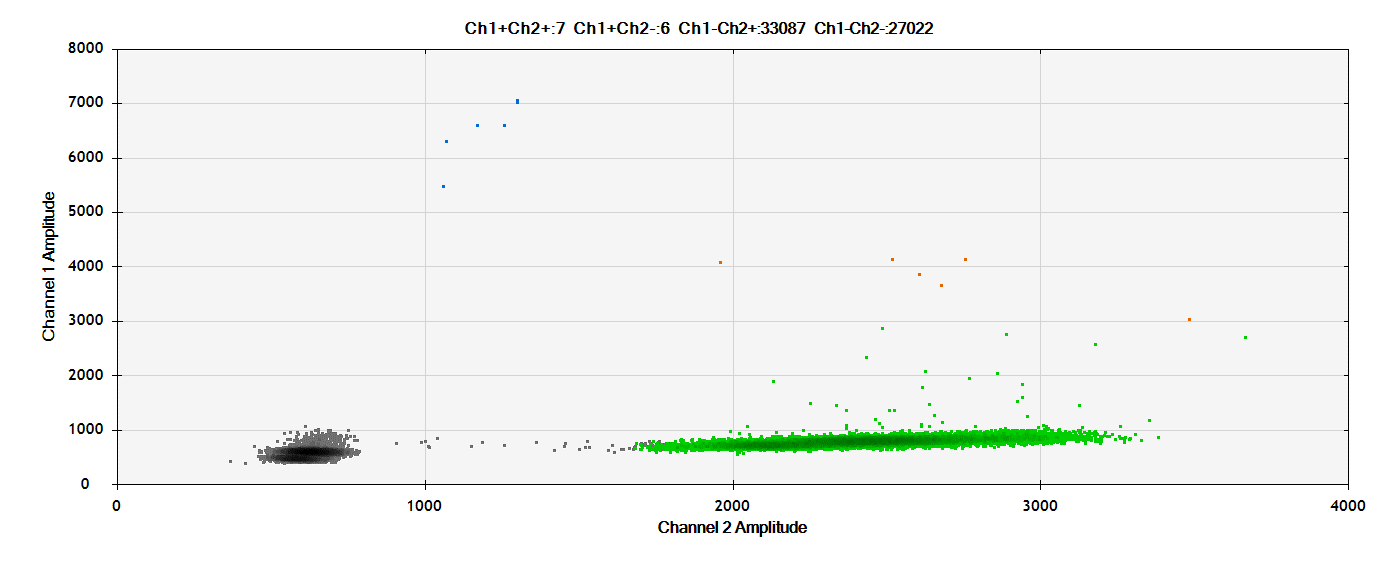


6 / X+4

AF=0.022%

HEX (Wt)

HI 1 (*PIGA*: c.718T>A, p.L271X)

HI 2 (*PIGA*: c.1280delT, p.I427Tfs*15)

Allelic frequencies (AFs) of the *PIGA* mutations determined at different time points in HI 1 and HI 2 are shown.
